# The Experiences and Reminiscences of Forty Years

**Published:** 1898-03

**Authors:** G. G. Roy

**Affiliations:** Emeritus Professor of Materia Medica and Therapeutics in Southern Medical College, Atlanta, Georgia


### THE EXPERIENCES AND REMINISCENCES OF FORTY YEARS.

BY G. G. ROY, M.D.,

Emeritus Professor of Materia Medica and Therapeutics in Southern Medical College, Atlanta.

In March, 1857, after receiving my medical education at the University of Virginia and the Jefferson Medical College of Philadelphia, I began the practice of medicine in my native State Virginia in connection with my father, who had been so engaged for a number of years.

Those were days when negroes were slaves and they received more careful medical skill and nursing than they ever will again. Because then they were the legal property of their owners and very valuable.

Though slaves, I never knew them treated otherwise than humanely and kindly, and there was a respect and love shown by them for their owners that was thoroughly tested during the late civil war, when in many instances they were the support and protection of the women and children throughout our Southland.

The negro is a superstitious being—was so then and not less so now—though it is natural to expect otherwise, in view of the large amount that is expended upon them yearly by the Southern people, whom they are now taught to look upon as their enemy.

In those days to be "tricked" or "conjured" by some other negro at enmity to them was an every-day occurrence; and to disabuse their minds of this fallacy by reassuring was a matter of impossibility.

I do not think Virginia negroes were more addicted to this superstition than those of other Southern States; but it is a fact that in those days numbers of Southern negroes and some of the ignorant whites adhered to that belief.

My first patient—when I landed from the steamboat plying the Rappahannock river between Baltimore, Md., and Fredericksburg, Va., from Philadelphia with my green case holding the then most precious legacy I desired, authorizing me by permission of the faculty of the Jefferson Medical College and the laws of the State of Pennsylvania, to go forth into the world to cure or kill—the first individual I met was old Sam Brown, colored freedman. Now Sam Brown was a noted character and had a history. At this period he was a very old man. His owner was a Frenchman and the captain of a brig plying between our southern ports and those of the North. Sam was one of his trusted hands and had become an expert sailor. After accumulating a fortune as a sea captain he located on the Rappahannock river in the county of my birth, and in addition to possessing magnificent lands and over a hundred negroes, he was engaged in the mercantile business on the river. Sam was all this time his trusted slave and friend.

In the war of 1812 the British landed a company of marines and set fire to this store. Everybody left the place but Sam Brown. He secreted himself in the marsh just behind the store. The British as soon as they applied the torch returned to their gunboats. Sam, taking advantage of this, before the fire had reached the "counting-room," rushed in, seized his master's account-book and a large sum of money where he fortunately knew it had been secreted, and thus saved several thousand dollars. His reward was his freedom given him by his master, Captain Janney.

Sam—as all worthy men should have—had a wife, dutiful and faithful, who was a slave, having another master. Her name was Bathias. Sam called her more endearingly, Thia. He *bought* her from her master, paying six hundred dollars, standard currency. The laws of Virginia allowed him to buy and own her as a slave just as slaves were bought and owned by whites. She then became both his wife and his slave. They were now quite old.

Uncle Sam, I say, was the first to greet me on landing. He said, "Mars Gus, de tell me you bin way out in de backwoods larning to be a doctor. Tell me, did you larn anything about curing 'conjuration' "? I told him I thought I would make a success of curing that troublesome disease, as I had been bending my energies to that end. He was greatly elated and comforted and said, "For de Lord sake, please come see Thia soon as you git home. Dat crater dun bin in bed for three years, conjured to def, and she gwine die sho if sum'ting ain't dun for her. Your pa and Dr. Smith I knows is good doctors, but dey won't hab nothing to do with Thia case dey say dare ain't no sich ting as 'conjuration.' Dat critter is gwine die, sho'." I promised him to call next day at 11 o'clock A.M., and true to promise I was there.

Lying on a bed in her cabin was the emaciated frame and haggard form of old Aunt Bathias Brown. She had taken little or no nourishment for a long time, and could not be induced to take it, unless brought out of the neighborhood, for fear her supposed enemy might put more "pizen" in it. This was my visit of prospection to ascertain her condition and see how the land lay before beginning my treatment.

I began by describing her feelings and symptoms myself, and wound up by telling her the *live things* in her, rapidly sapping her life away, collected into a large lump between her shoulders at 11 o'clock every day, and were a source of so much torment that she could not rest and could barely turn over for the lump, and they must be worms very much like fishing-worms, and some of them had grown very large after being hatched in her body, from the "pizen" of the conjurer. She clapped her hands and said, "Dat is de God's truf and I does believe you knows all 'bout conjuration."

I told her I would call next day promptly at 11 o'clock to try my hand at their extermination and her restoration.

Before the hour of starting on my visit to my patient I directed my office boy to go behind the smoke-house and dig up eight or ten fishing-worms and wrap them in a piece of paper and bring to me. He did so. For the purpose of somewhat shielding my conscience and for the wiser purpose of telling her I had not *seen* any live things that I might succeed in getting from her, I put the package in my vest pocket, never for once looking *to see* what was in it.

I reached her bedside at the appointed hour—told her she must be blindfolded (as that was a part of the treatment to make it a success), and lay flat upon her stomach so that I could readily get at the lump of live things between her shoulders. She consented, and placing her in this position I proceeded to cup her over the lump. She was so thin and emaciated that after using the scarificator three or four times, and a strong and new suction pump to exhaust the air from the cupping-glasses and draw out the blood, I only, after considerable effort, succeeded in getting about a teaspoonful of blood. With this I barely colored the water in the basin intended for washing my cupping-glass and also prevent her seeing the worms until she drained off the water. I took the paper containing the worms from my vest pocket and turning my head so as not to see them when emptied into the basin, and thereby making good my assertion to her that I had not seen a living thing, directed her to try to crawl out of her bed and take the basin and go to the fireplace, drain the water, and see for herself if there was anything in it; that I had not seen anything and could not swear there was, but I thought I had gotten something as the suction from the pump was so severe as to make me think it was drawing something out.

The old woman hobbled to the fireplace with the assistance of her cane, took a seat on a stool and began to drain off the water. Getting near the bottom she saw the worms wriggling, and, dropping the basin, began to shout and clap her hands, exclaiming, "I knowed them things was in me, and dey done near eat me up now, but praise God he done sent you here, way from de backwoods, to cure poor me."

Her joy was ecstatic. She put the worms into a bottle, corked it, tied a string around the neck of it, and hung it up in a secret place in her cabin.

She said at once she felt better and stronger, and "sorter hongry, too, for God knows I been feared to eat anything for months case I knowed dat nigger would pizen everything dat was cooked in dis house."

I continued the process of extracting the worms once a week for a month until I got a lot of tiny little worms, and assured her that they were the last; that I had destroyed the eggs and broken up the nest.

She began at once to improve, took nourishment (as I assured her it was safe to do so now, and if she got worse I would trick the nigger that had tricked her; this delighted her); gained strength and flesh, and in two months she was about her usual duties, hard at work, and lived for years after that in perfect health both of body and mind. During this treatment I gave no medicine, save the *medicina mentis.*

This, my first case, taught me the lesson that it is sometimes legitimate and necessary to use deception to the cure of such results of ignorance and superstition. It is an instance in which one is justified in doing evil that good may come.

(*To be continued.*)

